# Substandard and falsified systemic antiinfectives in Peru

**DOI:** 10.17843/rpmesp.2026.432.15577

**Published:** 2026-06-25

**Authors:** Carlos Huamaní-Pacsi, Roberto Torres Olivera

**Affiliations:** 1 Centro Nacional de Control de Calidad, Instituto Nacional de Salud, Lima, Peru.

**Keywords:** Substandard Drugs, Counterfeit Drugs, Drug Resistance Bacterial, Pharmacovigilance, Peru

## Abstract

Substandard and falsified (SF) systemic anti-infective medications (SAMs) detected by the National Health Authority of Peru between 2015 and 2023 were quantified and characterized. An observational, descriptive, and retrospective design was applied, based on test reports issued by the National Quality Control Center on the quality of SAIs, complemented with national evidence of antimicrobial resistance (AMR). Of 9799 pharmaceutical products evaluated, 1247 (12.7%) were SF; of these, 189 (15.2%) corresponded to SF-SAM, with falsified ones predominating (11.0%). The main groups were β-lactams (27.5%), sulfonamides/trimethoprim (26.5%), and quinolones (12.7%). 59.8% had informal origin, mostly from Lima (72%). The circulation of SF-SAM could promote selective pressure, the spread of AMR, and therapeutic failure.

## INTRODUCTION

Anti-infective or antimicrobial substances are natural or synthetic compounds capable of destroying, inhibiting, or delaying the multiplication of pathogenic microorganisms. According to their therapeutic use, they are classified as antibiotics, antivirals, antifungals, and antiparasitics ^1^.

It is estimated that there are more than a trillion microbial species; only a small fraction causes infection in humans, known as pathogens ^2^. All anti-infectives have the potential to induce antimicrobial resistance (AMR) when factors such as inadequate sanitary conditions, extensive use of anti-infectives in animals intended for human consumption, and critically, the administration of anti-infectives in subtherapeutic or poor-quality doses are present; these factors exert selective pressure that favors the survival and proliferation of resistant strains ^3^^,^^4^.

AMR constitutes a global challenge that affects countries of all income levels. Among its main consequences are the prolongation of hospital stays, the increase in healthcare costs for families, and the increase in mortality ^5^. In 2022, the World Health Organization (WHO) estimated that resistant infections caused at least 700,000 deaths per year, a figure that could rise to 10 million by 2050 ^1^.

Since 2017, the WHO has used the designation of substandard or falsified (SF) medical products to refer both to medicines that do not meet quality specifications, and to those whose identity, composition, and source have been falsified ^6^. The circulation of SF anti-infective medicines constitutes a critical challenge for public health, as it compromises therapeutic efficacy and promotes AMR. These medicines, by not complying with quality standards or containing fraudulent compositions, expose patients to insufficient doses, which facilitates the selection of resistant microorganisms and accelerates the spread of resistance mechanisms ^7^.

The lack of evidence integrating regulatory drug quality control data with antimicrobial resistance (AMR) epidemiological surveillance limits healthcare response capacity and hinders the formulation of evidence-based public policies. In this context, generating scientific information linking the quality of antimicrobials with AMR dynamics is essential to understand the associated risks, guide more effective regulatory interventions, and strengthen pharmacovigilance and microbiological surveillance.

This study aims to quantify and characterize SF systemic anti-infective medicines (SAM), investigated or seized by the National Health Authority of Peru and evaluated by the National Quality Control Center of the Instituto Nacional de Salud (INS) between 2015 and 2023. It also analyzes their implications as a health risk that favors the generation and spread of AMR in the country.

**KEY MESSAGES**

Motivation for the study. In Peru, a significant gap persists in understanding the role played by substandard and falsified medicines in the spread of antimicrobial resistance (AMR).

Main findings. It was found that 15.2% of substandard or falsified (SF) medicines were systemic anti-infectives (SAI), mainly β-lactams, sulfonamides/trimethoprim, and quinolones, widely used to treat common infections.

Public health implications. The integration of regulatory pharmacovigilance data with microbiological AMR surveillance will allow moving from late responses to evidence-based preventive interventions, optimizing resources and strengthening sustainable strategies.

## THE STUDY

An observational, descriptive, and retrospective study was conducted.

The study population consisted of all pharmaceutical products evaluated between January 2015 and December 2023 by the National Quality Control Center of the Instituto Nacional de Salud (CNCC-INS), a state entity responsible for drug quality control tests in Peru. The pharmaceutical products came from two main sources: the informal channel, consisting of medicines seized from illegal trade by the Dirección General de Medicamentos, Insumos y Drogas (DIGEMID) of the Ministry of Health, in coordination with the National Police of Peru and the Ministerio Público (Public Prosecutor's Office), which entered the CNCC-INS under the classification of «presumed falsified»; and the formal channel, comprising medicines surveyed in legal trade by DIGEMID, the Dirección de Redes Integradas de Salud (DIRIS) and the Direcciones Regionales de Salud (DIRESA), in pharmacies, drugstores, wholesalers, and pharmaceutical laboratory warehouses nationwide. These actions were motivated by complaints, sanitary risk criteria, cases of adverse reactions, or previous non-compliant results ^8^.

Information on the quality of SAM was obtained from the official test reports issued by the CNCC-INS, available in physical and digital format. Because these documents constitute internal technical information and are not publicly accessible, their use required institutional authorization from the CNCC-INS.

As this was a study based on secondary data from official CNCC-INS records, a sample was not defined; instead, all drug testing reports belonging to therapeutic group J (anti-infectives for systemic use), classified according to the Anatomical, Therapeutic, and Chemical (ATC) Classification System ^9^ and evaluated by the CNCC-INS, were included. Only those with a «non-compliant» result, identified as substandard and falsified, were considered. Incomplete reports or reports with deficient data that prevented a reliable analysis were excluded.

The classification of variables was performed according to the categories registered in the assay reports of the CNCC-INS. «Non-compliant» medicines were defined into two groups: substandard, which fail to meet quality standards due to deficient manufacturing or inadequate controls, and falsified, whose identity, composition, or origin have been deliberately or fraudulently altered. Characterization variables were considered, such as product name, year of the assay report, category according to the Anatomical, Therapeutic, and Chemical (ATC) Classification System, type of establishment (formal or informal), and geographical location.

Statistical analysis was performed using descriptive statistics with Microsoft Excel version 2016. Results were expressed in absolute frequencies and percentages, allowing for the assessment of the magnitude of the problem.

The study used a secondary database. Authorization was obtained from CNCC-INS to access the drug quality testing reports corresponding to the period 2015-2023. Approval from an institutional ethics committee was not required.

## FINDINGS

During the period 2015-2023, the CNCC-INS evaluated the quality of 9799 pharmaceutical products. A decreasing trend was observed from 2015 (n=2109) to 2021 (n=612), followed by an increase in 2022 (n=755) and 2023 (n=916). In this context, of the total products analyzed, 12.7% were classified as "non-compliant", and corresponded to SF medicines. Falsified medicines represented the highest proportion of non-compliant quality cases, with a minimum of 37 cases (5.9%) in 2020 and a maximum of 244 cases (39.9%) in 2021, the year that concentrated the highest relative incidence of falsified medicines. In contrast, substandard medicines were less frequent and showed a clear decreasing trend, decreasing from 70 cases (3.5%) in 2015 to only 5 cases (0.6%) in 2023 (table 1).

**Table 1:** Pharmaceutical products and SF medicines, reported by the National Quality Control Center of the Instituto Nacional de Salud, between 2015-2023.

Period (year)	Analyzed pharmaceutical products	Non-compliant medicines (all ATC categories)		
		Falsified	Substandard	Total
	n (%)	n (%)	n (%)	n (%)
2015	2109 (21.5)	113 (5.4)	70 (3.5)	183 (8.7)
2016	1688 (17.2)	112 (6.6)	45 (2.7)	157 (9.3)
2017	1299 (13.3)	59 (4.5)	19 (1.5)	78 (6.0)
2018	1104 (11.3)	178 (16.1)	12 (1.1)	190 (17.2)
2019	688 (7.0)	85 (12.4)	11 (1.6)	96 (14.0)
2020	628 (6.4)	37 (5.9)	7 (1.1)	44 (7.0)
2021	612 (6.2)	244 (39.9)	10 (1.6)	254 (41.5)
2022	755 (7.7)	104 (13.8)	10 (1.3)	114 (15.1)
2023	916 (9.3)	126 (13.8)	5 (0.6)	131 (14.3)
Total	9799	1058 (10.8)	189 (1.9)	1247 (12.7)

ATC: Anatomical, Therapeutic, and Chemical Classification.

Of the total non-compliant medicines (n=1247), 15.2% (n=189) corresponded to SF-SAM of therapeutic group J, with an annual variability between 7.5% in 2021 and 25.0% in 2019. Falsified SAM predominated, accumulating 137 cases (11.0%), with peaks in 2018 (24 cases) and 2019 (20 cases), and the lowest figure in 2020 (6 cases). Meanwhile, substandard SAM showed a sustained decreasing trend, registering 20 cases in 2015 until the absence of cases in 2023; in total, there were 52 cases (4.2%) in the 2015-2023 period (table 2).

**Table 2:** Substandard or falsified systemic anti-infectives reported by the National Quality Control Center of the Instituto Nacional de Salud, between 2015-2023.

Period (year)	Non-compliant medications (all the ATC categories)	Non-compliant systemic anti-infective medications (group J)	Falsified	Substandard
	n	n (%)	n (%)	n (%)
2015	183	41 (22.4)	21 (11.5)	20 (10.9)
2016	157	29 (15.8)	18 (11.5)	11 (7.0)
2017	78	9 (4.9)	7 (9,0)	2 (2.6)
2018	190	29 (15.8)	24 (12.6)	5 (2.6)
2019	96	24 (13.1)	20 (20.8)	4 (4.2)
2020	44	9 (4.9)	6 (13.6)	3 (6.8)
2021	254	19 (10.4)	15 (5.9)	4 (1.6)
2022	114	19 (10.4)	16 (14.0)	3 (2.6)
2023	131	10 (5.5)	10 (7.6)	0 (0.0)
Total	1247	189 (15.2)	137 (11.0)	52 (4.2)

ATC: Anatomical, Therapeutic and Chemical Classification.

The evaluation by therapeutic categories showed that the highest percentages were recorded in SAM from group J, subgroup J01 ^9^, beta-lactam antibacterials, penicillins (27.5%), sulfonamides/trimethoprim (26.5%), and antibacterial quinolones (12.7%), in all of them with a predominance of falsifications. In sulfonamides and trimethoprim, this finding was particularly marked, since 98% of the cases corresponded to falsifications. Other categories included macrolides, lincosamides, and streptogramins (8.5%), other beta-lactams other than penicillins (7.9%), tetracyclines (6.9%), direct-acting antivirals (5.3%), and other antibacterials (4.8%) (table 3).

**Table 3:** Substandard or falsified Systemic Anti-infectives by therapeutic subgroup reported by the National Quality Control Center of the Instituto Nacional de Salud, between 2015-2023.

Code	Therapeutic subgroup	Systemic anti-infective medications		
		Falsified	Substandard	Total
		n (%)	n (%)	n (%)
J01C	Beta-lactam Antibacterials, penicillins	33 (63.5)	19 (36.5)	52 (27.5)
J01E	Sulfonamides/trimethoprim	49 (98.0)	1 (2.0)	50 (26.5)
J01M	Antibacterial quinolones	18 (75.0)	6 (25.0)	24 (12.7)
J01F	Macrolides, lincosamides, and streptogramins	10 (62.5)	6 (37.5)	16 (8.5)
J01D	Other beta-lactam antibacterials	8 (53.3)	7 (46.7)	15 (7.9)
J01A	Tetracyclines	10 (76.9)	3 (23.1)	13 (6.9)
J05A	Direct-acting antivirals	5 (50.0)	5 (50.0)	10 (5.3)
J01X	Other antibacterials	4 (44.4)	5 (55.6)	9 (4.8)
	Total	137 (72.5)	52 (27.5)	189 (100.0)

Cases of substandard SAM were primarily related to failures in physical characteristics (40.4%), followed by deficiencies in content (17.3%), average weight (17.3%), identification tests (11.5%), dissolution (7.7%), and pH (5.8%). In falsified SAM, the most frequent irregularities were: presence of the correct active ingredient, but with a different content than declared (38.7%), products without an active ingredient (32.1%), and medicines with the correct active ingredient, but manufactured by a different laboratory than indicated on the label (29.2%) (table 4).

**Table 4:** Substandard or falsified systemic anti-infectives medications according to type of non-compliant assay, reported by the National Quality Control Center of the Instituto Nacional de Salud, between 2015-2023.

Category	Non-compliant test/irregularity	n (%)
Substandard (n=52)	Physical characteristics	21 (40.4)
	Content	9 (17.3)
	Average weight	9 (17.3)
	Identification	6 (11.5)
	Dissolution	4 (7.7)
	pH	3 (5.8)
Falsified (n=137)	Correct active ingredient, content different from that declared	53 (38.7)
	Without active ingredient	44 (32,1)
	Active ingredient and correct contents, different laboratory	40 (29,2)

59.8% of SF-SAM originated in informal trade, mainly *stands* or stalls in shopping centers (64.6%) and clandestine premises (16.8%). 40.2% originated in formal trade, highlighting private pharmacies (34.2%) and drugstores (28.9%). Geographically, Lima concentrated 72.0% of the cases, with a predominance of falsified medicines (75.7%), followed by Cajamarca (5.8%) and Junín (5.3%).

## DISCUSSION

The main findings of the study show that, between 2015 and 2023, 12.7% of the evaluated drugs were substandard and falsified. This study reveals that 15.2% of non-compliant drugs were SF- SAM, mainly β-lactams, sulfonamides/trimethoprim, and quinolones, with a predominance of falsifications (11.0%). Most originated in informal trade, although they were also detected in formal channels, with Lima concentrating the largest proportion.

SF systemic anti-infective drugs accounted for 15.2% of the total non-compliant drugs; this finding is consistent with previous reports in Latin America, where the circulation of falsified anti-infectives has been documented, particularly in informal markets ^10^. Recent studies indicate that the circulation of SF anti-infectives constitutes a critical factor in the generation of antimicrobial resistance, by exposing bacteria to subtherapeutic concentrations or ineffective treatments ^7^^,^^11^^,^^12^.

The therapeutic categories most affected by the circulation of SF-SAM corresponded to sulfonamides/trimethoprim, penicillins, and quinolones, in accordance with studies that indicate these antibacterials as the most vulnerable to falsification due to their high consumption and availability ^10^. 98% of sulfonamide/trimethoprim cases corresponded to falsifications, a critical finding due to their extended use in common infections, risk of therapeutic failure, and antimicrobial resistance ^7^^,^^13^.

Nearly 60% of SF-SAM came from informal trade, the main entry route. However, the finding of 40.2% in formal trade evidences weaknesses in the supply chain and the need to strengthen pharmacovigilance. The Lima region concentrated 72% of cases, with a predominance of falsified medicines (75.7%). Detection in other regions confirms a national problem, comparable with Latin American reports, where the circulation of falsified anti-infectives extends to peri-urban and rural areas ^10^^,^^13^. These findings should be interpreted in the context of the growing AMR crisis in Peru and how the circulation of SF-SAM would be related to this phenomenon ^14^^,^^15^^,^^16^.

Between 2015 and 2023, various studies on AMR were conducted in Peru, which identified multidrug-resistant strains such as *Acinetobacter baumannii, Klebsiella pneumoniae, Pseudomonas aeruginosa, Escherichia coli, Staphylococcus aureus* (MRSA)*, Enterococcus spp.* and *Salmonella/Shigella spp.*, with significant prevalence in hospitals in Lima, Callao, Loreto, Arequipa, and other regions ^17^^,^^18^. Furthermore, a study conducted in a Peruvian jungle hospital showed high resistance in gram-negative bacteria such as *Acinetobacter baumannii complex, Klebsiella pneumoniae ssp.* and *Pseudomonas aeruginosa* to β-lactams, particularly carbapenems such as imipenem and meropenem, with resistance rates exceeding 85% ^19^. Carbapenem resistance in *A. baumannii and K. pneumoniae* poses a direct threat to patients in intensive care units ^20^, while resistance to quinolones and sulfonamides/trimethoprim in *E. coli* affects the outpatient treatment of urinary tract infections ^21^. Additionally, there are high levels of *M. tuberculosis* resistance to antituberculosis drugs ^22^ and an increase in *Neisseria gonorrhoeae* resistance to ciprofloxacin ^23^. The INS, through the Vigilancia Integrada de la Resistencia Antimicrobiana (VIRAM) network in Peru, has reported in recent years a growing trend of resistance in clinically important pathogens such as *Escherichia coli* and *Klebsiella pneumoniae*, with high levels of resistance to third-generation cephalosporins and fluoroquinolones ^1^.

The chronological concurrence between both findings, SF-SAM and the sustained increase in clinically relevant multidrug-resistant strains, could be related; there is scientific evidence indicating that bacterial exposure to anti-infectives at subtherapeutic concentrations or absence of active principle favors selective pressure, resistance propagation and, consequently, therapeutic failure ^2^^,^^3^^,^^7^.

Therefore, pharmacovigilance and microbiological surveillance of AMR should be considered complementary strategies within a comprehensive public health approach. While VIRAM and the Global Antimicrobial Resistance and Use Surveillance System (GLASS) led by the WHO, provide microbiological data to guide clinical guidelines and rational use policies, the present study provides evidence on a critical link in the chain: the quality of SAM administered to patients. The integration of both sources of information would allow designing more effective interventions against AMR in the country.

This study has limitations that must be considered when interpreting the results. The information is restricted to medicines researched by health authorities in formal trade and seized in informal trade, which could underestimate the national magnitude, especially in regions with less oversight. Consequently, it is possible that the findings do not reflect the real magnitude of the problem at the national level. Likewise, the retrospective and descriptive design prevents the establishment of causal relationships or the estimation of population prevalences.

However, the findings acquire particular relevance because they correspond to the total of the SAM investigated or seized by the country's regulatory authority during the period 2015-2023. This characteristic confers robustness to the characterization of the identified products and allows clear trends to be evidenced, both in the progressive decrease of substandard SAM as well as in the persistence and predominance of falsified SAM.

In this regard, the results offer a solid empirical basis to strengthen regulatory policies, optimize the allocation of resources in health surveillance, and design integrated interventions that link microbiological surveillance with pharmacovigilance, to mitigate the impact of SF-SAM on AMR.

In conclusion, the results show that in Peru the circulation of SF-SAM could be associated with the selective pressure of resistant strains, the spread of AMR, and therapeutic failure. This finding is consistent with reports from the INS and the VIRAM network, corresponding to the 2015 - 2023 period, which documented high rates of resistance in clinically relevant pathogens and the CNCC-INS identified SF-SAM, in the same period. Collectively, the results constitute a key input to strengthen regulation and integrate pharmacovigilance with microbiological surveillance, as an essential strategy in the fight against AMR in the country.
