# Supplementary material for: Substandard and falsified systemic antiinfectives in Peru
Source: Rev Peru Med Exp Salud Publica. 2026 Jun 25;43(2):224–30. doi: 10.17843/rpmesp.2026.432.15577 (PMC13456916; doi:10.17843/rpmesp.2026.432.15577)
Supplement: Translation

# Translation: Antiinfecciosos de uso sistémico falsificados y subestándares en Perú

**Authors:** Carlos Huamaní-Pacsi, Roberto Torres Olivera

**Affiliations:** 1 Centro Nacional de Control de Calidad, Instituto Nacional de Salud, Lima, Perú.

**Keywords:** Medicamentos de Baja Calidad, Medicamentos Falsificados, Farmacorresistencia Bacteriana, Farmacovigilancia, Perú

## Abstract

Se cuantificó y caracterizó medicamentos antiinfecciosos de uso sistémico (AUS) subestándares y falsificados (SF) pesquisados por la Autoridad Sanitaria Nacional del Perú entre 2015 y 2023. Se aplicó un diseño observacional, descriptivo y retrospectivo basado en informes de ensayo emitidos por el Centro Nacional de Control de Calidad sobre calidad de los AUS, complementados con evidencia nacional de resistencia antimicrobiana (RAM). De 9799 productos farmacéuticos evaluados, 1247 (12,7%) fueron SF; de estos, 189 (15,2%) correspondieron a AUS SF, predominando los falsificados (11,0%). Los principales grupos fueron β-lactámicos (27,5%), sulfonamidas/trimetoprima (26,5%) y quinolonas (12,7%). El 59,8% tuvieron procedencia informal, mayormente de Lima (72%). La circulación de medicamentos AUS SF podría favorecer la presión selectiva, propagación de la RAM y el fracaso terapéutico.

## INTRODUCCIÓN

Las sustancias antiinfecciosas o antimicrobianas son compuestos naturales o sintéticos capaces de destruir, inhibir o retrasar la multiplicación de microorganismos patógenos. Según su uso terapéutico se clasifican como antibióticos, antivirales, antifúngicos y antiparasitarios ^1^.

Se estima la existencia de más de un billón de especies microbianas, solo una fracción reducida causa infección en humanos, conocidas como patógenos ^2^. Todos los antiinfecciosos tienen el potencial de inducir resistencia antimicrobiana (RAM) cuando se presentan factores como: condiciones inadecuadas de salubridad, uso extensivo de antiinfecciosos en animales destinados a consumo humano, y de manera crítica, la administración de antiinfecciosos en dosis subterapéuticas o de calidad deficiente; estos factores ejercen presión selectiva que favorece la supervivencia y proliferación de cepas resistentes ^3^^,^^4^.

La RAM constituye un desafío global que afecta a países de todos los niveles de ingresos. Entre sus principales consecuencias se encuentran la prolongación de estancias hospitalarias, el aumento de costos de atención sanitaria para las familias y el incremento de la mortalidad ^5^. En 2022, la Organización Mundial de la Salud (OMS) estimó que las infecciones resistentes ocasionaban al menos 700 000 muertes al año, cifra que podría ascender a 10 millones para el 2050 ^1^.

La OMS utiliza desde el 2017 la denominación de productos médicos de calidad subestándar o falsificados (SF) para referirse tanto a los medicamentos que no cumplen con las especificaciones de calidad, como aquellos cuya identidad, composición y fuente han sido falsificadas ^6^. La circulación de medicamentos antiinfecciosos SF constituye un desafío crítico para la salud pública, ya que compromete la eficacia terapéutica y favorece la RAM. Estos medicamentos, al no cumplir con estándares de calidad o contener composiciones fraudulentas, exponen a los pacientes a dosis insuficientes, lo que facilita la selección de microorganismos resistentes y acelera la propagación de mecanismos de resistencia ^7^.

La falta de evidencia que integre los datos regulatorios de control de calidad de medicamentos con la vigilancia epidemiológica de la RAM limita la capacidad de respuesta sanitaria y dificulta la formulación de políticas públicas basadas en evidencia. En este contexto, generar información científica que vincule la calidad de los antimicrobianos con la dinámica de la RAM es esencial para comprender los riesgos asociados, orientar intervenciones regulatorias más efectivas y fortalecer la farmacovigilancia y la vigilancia microbiológica.

Este estudio tiene como objetivo cuantificar y caracterizar los medicamentos antiinfecciosos de uso sistémico (AUS) SF, pesquisados o incautados por la Autoridad Sanitaria Nacional del Perú y evaluados por el Centro Nacional de Control de Calidad del Instituto Nacional de Salud (INS) entre 2015 y 2023. También, analiza sus implicancias como un riesgo sanitario que favorece la generación y propagación de la RAM en el país.

**MENSAJES CLAVE**

Motivación para realizar el estudio. En el Perú, persiste una brecha significativa en la comprensión del papel que desempeñan los medicamentos subestándares y falsificados en la propagación de la resistencia antimicrobiana (RAM).

Principales hallazgos. Se halló que el 15,2% de los medicamentos subestándares o falsificados (SF) fueron antiinfecciosos de uso sistémico (AUS), principalmente β-lactámicos, sulfonamidas/trimetoprima y quinolonas, muy usadas para tratar infecciones comunes.

Implicancias en salud pública. La integración de datos regulatorios de la farmacovigilancia con la vigilancia microbiológica de la RAM permitirá pasar de respuestas tardías a intervenciones preventivas basadas en evidencia, optimizando recursos y fortaleciendo estrategias sostenibles.

## EL ESTUDIO

Se realizó un estudio observacional, descriptivo y retrospectivo.

La población de estudio estuvo conformada por todos los productos farmacéuticos evaluados entre enero del 2015 y diciembre del 2023 por el Centro Nacional de Control de Calidad del Instituto Nacional de Salud (CNCC-INS), entidad estatal responsable de los ensayos de control de calidad de medicamentos en el Perú. Los productos farmacéuticos procedían de dos fuentes principales: el canal informal, constituido por medicamentos incautados del comercio ilegal por la Dirección General de Medicamentos, Insumos y Drogas (DIGEMID) del Ministerio de Salud, en coordinación con la Policía Nacional del Perú y el Ministerio Público, los cuales ingresaron al CNCC-INS bajo la clasificación de «presuntamente falsificados»; y el canal formal, integrado por medicamentos pesquisados en el comercio legal por la DIGEMID, la Dirección de Redes Integradas de Salud (DIRIS) y las Direcciones Regionales de Salud (DIRESA), en farmacias, boticas, droguerías y almacenes de laboratorios farmacéuticos a nivel nacional. Estas acciones fueron motivadas por denuncias, criterios de riesgo sanitario, casos de reacciones adversas o resultados no conformes previos ^8^.

La información sobre la calidad de los medicamentos AUS se obtuvo de los informes oficiales de ensayo emitidos por el CNCC-INS, disponibles en formato físico y digital. Debido a que estos documentos constituyen información técnica de uso interno y no son de acceso público, su utilización requirió autorización institucional del CNCC-INS.

Al tratarse de un estudio basado en datos secundarios provenientes de registros oficiales del CNCC-INS, no se definió una muestra, sino que se incluyó la totalidad de los informes de ensayo de medicamentos pertenecientes al grupo terapéutico J (antiinfecciosos para uso sistémico), clasificados según el Sistema de Clasificación Anatómica, Terapéutica y Química (ATC) ^9^ y evaluados por el CNCC-INS. Se consideraron únicamente aquellos con resultado «no conforme», identificados como subestándares y falsificados. Se excluyeron los informes incompletos o con datos deficientes que impidieran un análisis confiable.

La clasificación de las variables se realizó conforme a las categorías registradas en los informes de ensayo del CNCC-INS. Los medicamentos «no conformes» se definieron en dos grupos: subestándar, que incumplen normas de calidad por fabricación deficiente o controles inadecuados, y falsificados, cuya identidad, composición o procedencia han sido alteradas de manera deliberada o fraudulenta. Se consideraron variables de caracterización como nombre del producto, año del informe de ensayo, categoría según el Sistema de Clasificación Anatómica, Terapéutica y Química (ATC), tipo de establecimiento (formal o informal) y ubicación geográfica.

El análisis estadístico se realizó mediante estadística descriptiva utilizando Microsoft Excel versión 2016. Los resultados se expresaron en frecuencias absolutas y porcentajes, permitiendo dimensionar la magnitud del problema.

El estudio utilizó una base de datos secundaria. Se obtuvo autorización del CNCC-INS para acceder a los informes de ensayo de calidad de medicamentos correspondiente al periodo 2015-2023. No se requirió aprobación de un comité de ética institucional.

## HALLAZGOS

Durante el periodo 2015-2023, el CNCC-INS evaluó la calidad de 9799 productos farmacéuticos. Se evidenció una tendencia decreciente desde 2015 (n=2109) hasta 2021 (n=612), seguido por un incremento en 2022 (n=755) y 2023 (n=916). En este contexto, del total de productos analizados, el 12,7% fue clasificado como «no conforme», y correspondió a medicamentos SF. Los medicamentos falsificados representaron la mayor proporción de los casos de calidad no conforme, con un mínimo de 37 casos (5,9%) en el 2020 y un máximo de 244 casos (39,9%) en el 2021, año que concentró la mayor incidencia relativa de medicamentos falsificados. En contraste, los medicamentos subestándares fueron menos frecuentes y mostraron una clara tendencia decreciente, disminuyendo de 70 casos (3,5%) en el 2015 a solo 5 casos (0,6%) en el 2023 (tabla 1).

**Tabla 1:** Productos farmacéuticos y medicamentos SF, reportados por el Centro Nacional de Control de Calidad del Instituto Nacional de Salud, entre el 2015-2023.

Periodo (año)	Productos farmacéuticos analizados	Medicamentos no conformes (todas las categorías ATC)		
		Falsificados	Subestándar	Total
	n (%)	n (%)	n (%)	n (%)
2015	2109 (21,5)	113 (5,4)	70 (3,5)	183 (8,7)
2016	1688 (17,2)	112 (6,6)	45 (2,7)	157 (9,3)
2017	1299 (13,3)	59 (4,5)	19 (1,5)	78 (6,0)
2018	1104 (11,3)	178 (16,1)	12 (1,1)	190 (17,2)
2019	688 (7,0)	85 (12,4)	11 (1,6)	96 (14,0)
2020	628 (6,4)	37 (5,9)	7 (1,1)	44 (7,0)
2021	612 (6,2)	244 (39,9)	10 (1,6)	254 (41,5)
2022	755 (7,7)	104 (13,8)	10 (1,3)	114 (15,1)
2023	916 (9,3)	126 (13,8)	5 (0,6)	131 (14,3)
Total	9799	1058 (10,8)	189 (1,9)	1247 (12,7)

ATC: Clasificación Anatómica, Terapéutica y Química.

Del total de medicamentos no conformes (n=1247), el 15,2 % (n=189) correspondió a medicamentos AUS SF del grupo terapéutico J, con una variabilidad anual entre 7,5 % en 2021 y 25,0 % en 2019. Los medicamentos AUS falsificados predominaron, ya que acumularon 137 casos (11,0 %), con picos en 2018 (24 casos) y el 2019 (20 casos), y la cifra más baja el 2020 (6 casos). Por su parte, los medicamentos AUS subestándares mostraron una tendencia decreciente sostenida, registrando 20 casos en 2015 hasta la ausencia de casos en 2023, en total fueron 52 casos (4,2 %) en el periodo 2015-2023 (tabla 2).

**Tabla 2:** Antiinfecciosos de uso sistémico subestándares o falsificados reportados por el Centro Nacional de Control de Calidad del Instituto Nacional de Salud, entre el 2015-2023.

Periodo (año)	Medicamentos no conformes (todas las categorías ATC)	Antiinfecciosos de uso sistémico no conformes (grupo J)	Falsificados	Subestándar
	n	n (%)	n (%)	n (%)
2015	183	41 (22,4)	21 (11,5)	20 (10,9)
2016	157	29 (15,8)	18 (11,5)	11 (7,0)
2017	78	9 (4,9)	7 (9,0)	2 (2,6)
2018	190	29 (15,8)	24 (12,6)	5 (2,6)
2019	96	24 (13,1)	20 (20,8)	4 (4,2)
2020	44	9 (4,9)	6 (13,6)	3 (6,8)
2021	254	19 (10,4)	15 (5,9)	4 (1,6)
2022	114	19 (10,4)	16 (14,0)	3 (2,6)
2023	131	10 (5,5)	10 (7,6)	0 (0,0)
Total	1247	189 (15,2)	137 (11,0)	52 (4,2)

ATC: Clasificación Anatómica, Terapéutica y Química.

La evaluación por categorías terapéuticas evidenció que los porcentajes más elevados se registraron en los medicamentos AUS del grupo J, subgrupo J01 ^9^, antibacterianos betalactámicos, penicilinas (27,5%), sulfonamidas/trimetoprima (26,5%) y quinolonas antibacterianas (12,7%), en todas ellas con predominio de falsificados. En las sulfonamidas y trimetoprima este hallazgo fue particularmente marcado, ya que el 98% de los casos correspondió a falsificaciones. Otras categorías incluyeron macrólidos, lincosamidas y estreptograminas (8,5%), otros betalactámicos distintos de penicilinas (7,9%), tetraciclinas (6,9%), antivirales de acción directa (5,3%) y otros antibacterianos (4,8%) (tabla 3).

**Tabla 3:** Antiinfecciosos de Uso Sistémico subestándares o falsificados según subgrupo terapéutico reportado por el Centro Nacional de Control de Calidad del Instituto Nacional de Salud, entre el 2015-2023.

Código	Subgrupo terapéutico	Medicamentos antiinfecciosos de uso sistémico		
		Falsificados	Subestándar	Total
		n (%)	n (%)	n (%)
J01C	Antibacterianos betalactámicos, penicilinas	33 (63,5)	19 (36,5)	52 (27,5)
J01E	Sulfonamidas/trimetoprima	49 (98,0)	1 (2,0)	50 (26,5)
J01M	Quinolonas antibacterianas	18 (75,0)	6 (25,0)	24 (12,7)
J01F	Macrólidos, lincosamidas y estreptograminas	10 (62,5)	6 (37,5)	16 (8,5)
J01D	Otros antibacterianos betalactámicos	8 (53,3)	7 (46,7)	15 (7,9)
J01A	Tetraciclinas	10 (76,9)	3 (23,1)	13 (6,9)
J05A	Antivirales de acción directa	5 (50,0)	5 (50,0)	10 (5,3)
J01X	Otros antibacterianos	4 (44,4)	5 (55,6)	9 (4,8)
	Total	137 (72,5)	52 (27,5)	189 (100,0)

Los casos de medicamentos AUS subestándares se relacionaron principalmente con fallas en caracteres físicos (40,4%), seguidas de deficiencias en el contenido (17,3%), peso promedio (17,3%), pruebas de identificación (11,5%), disolución (7,7%) y pH (5,8%). En los medicamentos AUS falsificados, las irregularidades más frecuentes fueron: presencia de principio activo correcto, pero con contenido diferente al declarado (38,7%), productos sin principio activo (32,1%) y medicamentos con principio activo correcto, pero fabricados por un laboratorio distinto al rotulado (29,2%) (tabla 4).

**Tabla 4:** Antiinfecciosos de uso sistémico subestándares o falsificados según tipo de ensayo no conforme, reportados por el Centro Nacional de Control de Calidad del Instituto Nacional de Salud, entre el 2015-2023.

Categoría	Ensayo no conforme/irregularidad	n (%)
Subestándar (n=52)	Caracteres físicos	21 (40,4)
	Contenido	9 (17,3)
	Peso promedio	9 (17,3)
	Identificación	6 (11,5)
	Disolución	4 (7,7)
	pH	3 (5,8)
Falsificados (n=137)	Principio activo correcto, contenido diferente al declarado	53 (38,7)
	Sin principio activo	44 (32,1)
	Principio activo y contenido correctos, distinto laboratorio	40 (29,2)

El 59,8% de los AUS SF se originó en el comercio informal, principalmente *stands* o puestos en centros comerciales (64,6%) e inmuebles clandestinos (16,8%). El 40,2% se originó en el comercio formal, destacando boticas privadas (34,2%) y droguerías (28,9%). Geográficamente, Lima concentró el 72,0% de los casos, con predominio de medicamentos falsificados (75,7%), seguido de Cajamarca (5,8%) y Junín (5,3%).

## DISCUSIÓN

Los principales hallazgos del estudio evidencian que, entre el 2015 y 2023, el 12,7% de los medicamentos evaluados fueron subestándares y falsificados. Este estudio revela que el 15,2% de los medicamentos no conformes fueron medicamentos AUS SF, principalmente β-lactámicos, sulfonamidas/trimetoprima y quinolonas, con predominio de falsificaciones (11,0%). La mayoría se originó en el comercio informal, aunque también se detectaron en canales formales, con Lima concentrando la mayor proporción.

Los medicamentos antiinfecciosos de uso sistémico SF representaron el 15,2 % del total de medicamentos no conformes, este hallazgo es consistente con reportes previos en América Latina, donde se ha documentado circulación de antiinfecciosos falsificados, particularmente en mercados informales ^10^. Estudios recientes señalan que la circulación de antiinfecciosos SF constituye un factor crítico en la generación de resistencia antimicrobiana, al exponer a las bacterias a concentraciones subterapéuticas o a tratamientos ineficaces ^7^^,^^11^^,^^12^.

Las categorías terapéuticas más comprometidas por la circulación de medicamentos AUS SF correspondieron a sulfonamidas/trimetoprima, penicilinas y quinolonas, en concordancia con estudios que señalan a estos antibacterianos como los más vulnerables a la falsificación por su alto consumo y disponibilidad ^10^. El 98% de los casos de sulfonamidas/trimetoprima correspondió a falsificaciones, hallazgo crítico por su uso extendido en infecciones comunes, riesgo de fracaso terapéutico y resistencia antimicrobiana ^7^^,^^13^.

Cerca del 60% de medicamentos AUS SF provino del comercio informal, principal vía de ingreso. Sin embargo, el hallazgo del 40,2% en el comercio formal evidencia debilidades en la cadena de suministro y necesidad de fortalecer la farmacovigilancia. La región Lima concentró el 72% de casos, con predominio de medicamentos falsificados (75,7%). La detección en otras regiones confirma un problema nacional, comparable con reportes latinoamericanos, donde la circulación de antiinfecciosos falsificados se extiende a áreas periurbanas y rurales ^10^^,^^13^. Estos hallazgos deben interpretarse en el contexto de la creciente crisis de RAM en el Perú y como la circulación de los medicamentos AUS SF estaría relacionado a este fenómeno ^14^^,^^15^^,^^16^.

Entre el 2015 y 2023, se realizaron diversos estudios sobre RAM en el Perú, los cuales identificaron cepas multirresistentes como *Acinetobacter baumannii, Klebsiella pneumoniae, Pseudomonas aeruginosa, Escherichia coli, Staphylococcus aureus* (MRSA)*, Enterococcus spp.* y *Salmonella/Shigella spp.*, con prevalencia significativa en hospitales de Lima, Callao, Loreto, Arequipa y otras regiones ^17^^,^^18^. Asimismo, un estudio realizado en un hospital de la selva peruana, evidencio una elevada resistencia en bacterias gramnegativas como *Acinetobacter baumannii complex, Klebsiella pneumoniae ssp.* y *Pseudomonas aeruginosa* frente a β-lactámicos, particularmente carbapenémicos como imipenem y meropenem, con tasas de resistencia superiores al 85% ^19^. La resistencia a carbapenémicos en *A. baumannii y K. pneumoniae* representa una amenaza directa para pacientes en unidades de cuidados intensivos ^20^, mientras que la resistencia a quinolonas y sulfonamidas/trimetoprima en *E. coli* afecta el tratamiento ambulatorio de infecciones urinarias ^21^. Adicionalmente, existe elevados niveles de resistencia de *M. tuberculosis* a los medicamentos antituberculosos ^22^^)^ e incremento de la resistencia de *Neisseria gonorrhoeae* al ciprofloxacino ^23^. El INS, a través de la red de Vigilancia Integrada de la Resistencia Antimicrobiana (VIRAM) en Perú, ha reportado en los últimos años una tendencia creciente de resistencia en patógenos de importancia clínica como *Escherichia coli* y *Klebsiella pneumoniae*, con niveles elevados de resistencia a cefalosporinas de tercera generación y fluoroquinolonas ^1^.

La concurrencia cronológica entre ambos hallazgos, medicamentos AUS SF y el incremento sostenido de cepas multirresistentes clínicamente relevantes podría estar relacionada; existe evidencia científica que indica que la exposición bacteriana a antiinfecciosos en concentraciones subterapéuticas o ausencia de principio activo favorece la presión selectiva, propagación de resistencia y, en consecuencia, el fracaso terapéutico ^2^^,^^3^^,^^7^.

Por tanto, la farmacovigilancia y la vigilancia microbiológica de la RAM deben considerarse estrategias complementarias dentro de un enfoque integral de salud pública. Mientras VIRAM y el Sistema Mundial de Vigilancia del Uso y la Resistencia a los Antimicrobianos (GLASS) liderados por la OMS, proporcionan datos microbiológicos para orientar guías clínicas y políticas de uso racional, el presente estudio aporta evidencia sobre un eslabón crítico de la cadena: la calidad de los medicamentos AUS que se administra a los pacientes. La integración de ambas fuentes de información permitiría diseñar intervenciones más efectivas frente a la RAM en el país.

Este estudio tiene limitaciones que deben considerarse al interpretar los resultados. La información se restringe a medicamentos pesquisados por las autoridades sanitarias en el comercio formal e incautados en el informal, lo que podría subestimar la magnitud nacional, especialmente en regiones con menor fiscalización. Por consiguiente, es posible que los hallazgos no reflejen la magnitud real del problema a nivel nacional. Asimismo, el diseño retrospectivo y descriptivo impide establecer relaciones causales o estimar prevalencias poblacionales.

No obstante, los hallazgos adquieren particular relevancia porque corresponden al total de los medicamentos AUS pesquisados o incautados por la autoridad fiscalizadora del país durante el periodo 2015-2023. Esta característica confiere robustez a la caracterización de los productos identificados y permite evidenciar tendencias claras, tanto en la disminución progresiva de los medicamentos AUS subestándares como en la persistencia y predominio de los medicamentos AUS falsificados.

En este sentido, los resultados ofrecen una base empírica sólida para fortalecer políticas regulatorias, optimizar la asignación de recursos en vigilancia sanitaria y diseñar intervenciones integradas que articulen la vigilancia microbiológica con la farmacovigilancia, para mitigar el impacto de los medicamentos AUS SF en la RAM.

En conclusión, los resultados evidencian que en el Perú la circulación de medicamentos AUS SF podría estar asociado con la presión selectiva de cepas resistentes, la propagación de la RAM y el fracaso terapéutico. Este hallazgo, es consistente con reportes del INS y de la red de VIRAM, correspondientes al periodo 2015 - 2023, que documentaron altas tasas de resistencia en patógenos clínicamente relevantes y el CNCC-INS identificó medicamentos AUS SF, en el mismo periodo. En conjunto, los resultados constituyen un insumo clave para fortalecer la regulación e integrar la farmacovigilancia con la vigilancia microbiológica, como estrategia esencial en lucha contra la RAM en el país.
